# Clinical performance and cost-effectiveness of low-shrinkage giomer resin composite versus resin-modified glass ionomer in cervical carious lesions: a 12-month randomized controlled trial

**DOI:** 10.1186/s12903-025-06594-y

**Published:** 2025-08-06

**Authors:** Menna El Ghamrawy, Dina Kamal, Heba Hamza

**Affiliations:** 1 Conservative Dentistry Department, Faculty of Dentistry, Cairo University, Cairo, Egypt; 2 Conservative Dentistry Department, School of Dentistry, Newgiza University, Giza, Egypt

**Keywords:** Class V cavities, Cervical carious lesions, Giomer technology, Low-shrinkage giomer resin composite, Resin-modified glass ionomer, Cost-effectiveness, Revised FDI criteria

## Abstract

**Background:**

Cervical carious lesions present a clinical challenge due to several factors and require restorative materials with optimal performance. This trial evaluated the clinical performance and cost-effectiveness of low-shrinkage giomer resin composite (LS-GRC) compared to resin-modified glass ionomer (RMGI) for restoring cervical carious lesions over a 12-month period.

**Participants and methods:**

A total of 56 class V cavities were randomly assigned to two groups (*n* = 28). Intervention group received LS-GRC (Beautifil II LS, Shofu Dental Corporation, Kyoto, Japan), and control group received RMGI (Fuji II LC, GC Corporation, Tokyo, Japan). Restorations were evaluated using revised FDI criteria at baseline, 6, and 12 months. Data were statistically analyzed with a significance level of *P* ≤ 0.05. Intergroup comparisons were assessed with Chi-squared test, while intragroup comparisons were assessed with Cochran’s Q test. Incremental cost-effectiveness ratio (ICER) and cost per success ratio (CPSR) were used for cost-effectiveness analysis.

**Results:**

After 12 months, intergroup comparisons revealed no significant differences for all outcomes (*P* > 0.05), except for surface luster and texture, which favored LS-GRC (*P* < 0.05). Intragroup comparisons revealed no significant differences within LS-GRC group (*P* > 0.016), while within RMGI group, significant differences were observed for surface luster and texture after 12 months (*P* < 0.016). ICER analysis showed that the cost for each additional 1% improvement in clinical outcomes with LS-GRC was approximately 1.6 times higher than that of RMGI. The CPSR for LS-GRC was 4.6% lower than that of RMGI, indicating that, despite its higher initial cost, LS-GRC provided comparable clinical performance, with significantly improved esthetic surface quality and favorable cost-effectiveness over the 12-month period.

**Conclusions:**

LS-GRC and RMGI exhibited comparable performance and were clinically acceptable after 12 months.

**Clinical relevance:**

Low-shrinkage giomer resin composite offers bioactivity, superior restoration integrity, and excellent esthetics, helping achieve optimal cervical restorations with improved clinical success, durability, and sustained cost-effectiveness.

**Trial registration:**

https://clinicaltrials.gov/, (NCT05930548), 30–06–2023.

**Graphical Abstract:**

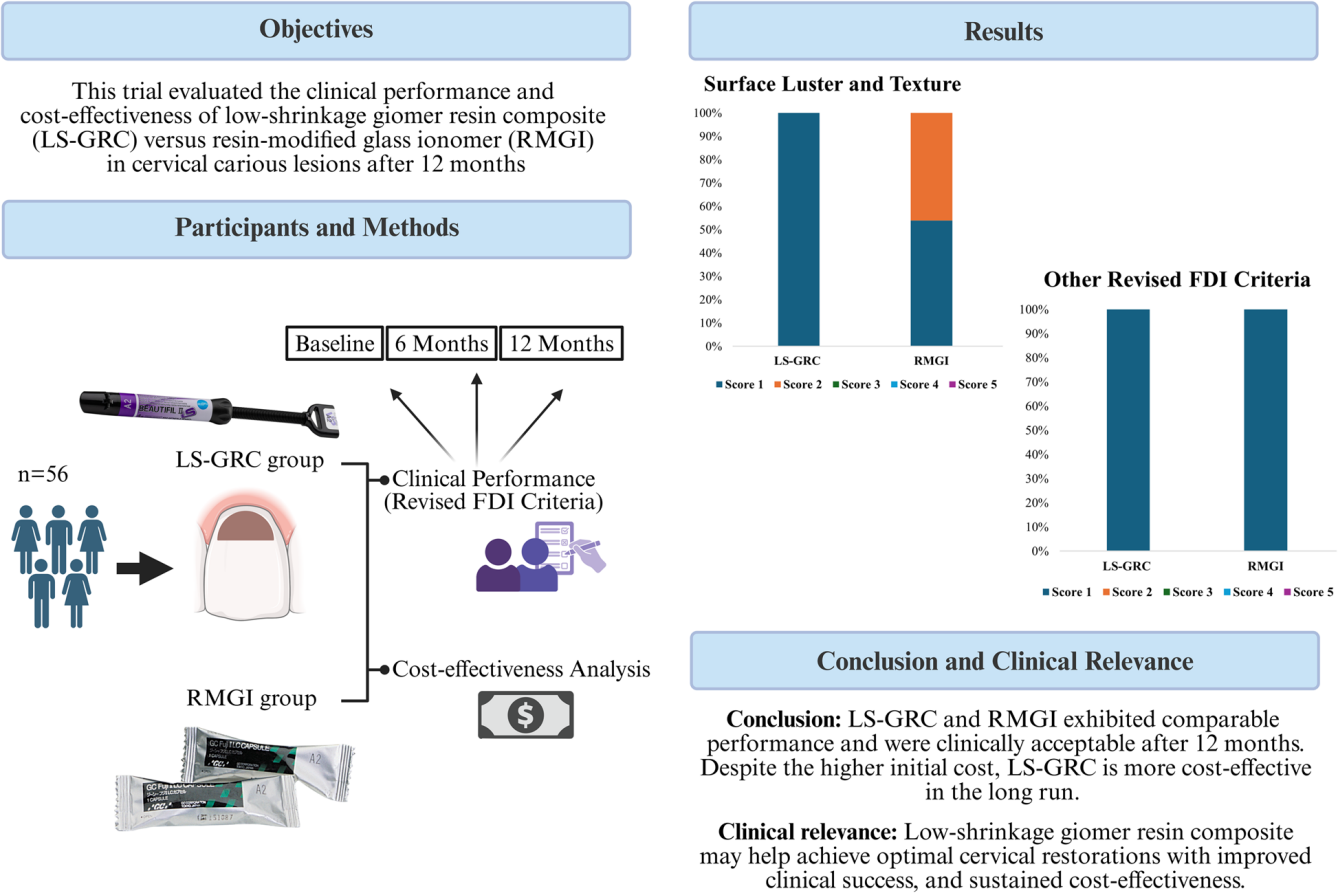

## Introduction

Caries management is technically demanding and requires consideration of various factors, including the adoption of minimally invasive techniques, the use of biocompatible restorative materials, aesthetic considerations, and patient-specific factors. All these elements are essential for achieving both clinical success and patient satisfaction [1].

Cervical carious lesions are frequently encountered in clinical practice, posing a significant challenge due to limited or absent enamel margins, which complicate sealing and contribute to microleakage [2]. A major cause of failure in cervical restorations is compromised bonding to dentin/cementum [3]. Additionally, the high configuration factor (C-factor) of cervical cavities, coupled with dimensional changes in restorative materials due to polymerization shrinkage, thermal contraction, water sorption, and flexural stresses, further complicate restoration outcomes [4, 5].

Recent advancements in minimally invasive dentistry have shifted toward restorative biomaterials capable of ion-release. Unlike inert materials, contemporary materials should exhibit anti-demineralizing and remineralizing properties to ensure long-term stability, while also withstanding occlusal loads, thermal variations, and enzymatic degradation [6]. Accordingly, materials such as resin-modified glass ionomer (RMGI) and giomers have been introduced [7].

The popularity of RMGIs in restoring cervical cavities is attributed to their unique bonding to tooth structure, biological sealing of dentin, biocompatibility, fluoride release and recharge, and superior mechanical properties [8, 9]. However, their use may be limited in esthetically critical areas due to their chemical composition and polishability, which can impact color stability [10].

Giomers have been introduced as a true hybrid of glass ionomers and resin composites by incorporating surface pre-reacted glass ionomer (S-PRG) fillers into the resin matrix. This combination provides protection against secondary caries, along with functional and esthetic benefits [4, 11]. Among the latest developments, low-shrinkage giomer resin composites (LS-GRC) have gained recognition for their reduced volumetric polymerization shrinkage, which mitigates shrinkage stresses [12]. Additionally, LS-GRCs offer improved mechanical and optical properties, enhanced gloss retention, and increased wear resistance [2].

Cost is a critical factor in treatment decisions, as both healthcare providers and patients must consider financial implications alongside clinical outcomes. Given that treatment costs are a significant concern, conducting a cost-effectiveness analysis is essential to evaluate the value of different treatment options. A treatment modality that may initially appear costly but demonstrates long-term clinical success without complications is considered cost-effective [13].

Although a substantial body of literature exists on non-carious cervical lesions, research specifically addressing the restoration of cervical carious lesions remains limited [14]. In this clinical trial, the main objective was to compare the clinical performance and cost-effectiveness of LS-GRC and RMGI in the restoration of cervical carious lesions. Specifically, this trial aimed to evaluate the clinical outcomes and economic impact of these materials over a 12-month period. The null hypothesis tested was that there would be no difference between LS-GRC and RMGI in terms of clinical outcomes and cost-effectiveness in the restoration of cervical carious lesions after 12 months.

## Participants and methods

### Ethics approval and trial registration

All procedures involving participants adhered to the ethical standards of the 1975 Helsinki Declaration and obtained approval from the Research Ethics Committee, Faculty of Dentistry, Cairo University (ID: 10723). The study was registered in ClinicalTrials.gov registry (https://clinicaltrials.gov/, ID: NCT05930548) on 23 June 2023. The Consolidated Standards of Reporting Trials (CONSORT 2025) statement was followed to design the study.

### Sample size calculation

Sample size was calculated using G*Power version 3.1.9.2 for Windows, based on a previous study [10], reporting a 68.7% success rate for marginal discoloration of class V cavities (Scores A and B) restored with resin-modified glass ionomer. A two-tailed Z-test was conducted to compare two independent proportions, using a 5% alpha level and 80% power. Each group required a sample size of 23 to identify a 30% difference. To account for potential dropouts, the sample size was increased by 20%, resulting in 28 teeth per group.

### Study design and setting

This is a randomized, triple-blind, parallel-group trial with a 1:1 allocation ratio, conducted over 12 months using a non-inferiority framework. The trial was carried out at the Conservative Dentistry clinics, Faculty of Dentistry, Cairo University, Egypt, from September 2023 to January 2025. Fifty-six participants with cervical carious cavities were randomly allocated to two groups (*n* = 28). The intervention group was restored with LS-GRC, and the control group was restored with RMGI. Clinical performance was evaluated at baseline, 6 months, and 12 months using the revised World Dental Federation (FDI) criteria. Participants were fully briefed on the study and provided written informed consent. Patient history, caries risk profile, and clinical examinations were documented in diagnostic records. Eligible participants underwent scaling and polishing, and any existing dental issues were addressed prior to the trial's initiation. Participants were instructed on effective toothbrushing and flossing techniques, with these practices reinforced at every follow-up visit. No changes were made to the trial protocol after it commenced.

### Eligibility criteria

Inclusion criteria included male or female participants aged 22 to 45 years with supragingival class V cavities in maxillary anterior teeth, not extending beyond the cementoenamel junction (CEJ) and classified as ICDAS code (4); teeth with normal pulp vitality and healthy periodontal status; patients identified as moderate or high caries-risk based on the American Dental Association (ADA) assessment model; and those with general good health. The exclusion criteria comprised serious or major health conditions; bruxism/clenching habits; TMJ dysfunction; signs of pulp or periapical pathology; active periodontal disease; xerostomia; smoking; pregnancy; drug addiction; or conditions that could interfere with study compliance.

### Randomization, sequence generation, and allocation concealment

Fifty-six eligible participants (25 males, 31 females, mean age: 32.8 ± 7.94 years) were randomly allocated to two groups. Simple randomization was performed by generating numbers from 1 to 56 using a computer-generated sequence (https://www.random.org/). Participants numbered 1–28 were allocated to the intervention group, and those numbered 29–56 to the control group. The allocation sequence was enclosed in sequentially numbered, concealed and secured envelopes by an individual uninvolved in the study. Each envelope was opened only after the participant enrollment, ensuring allocation concealment. The researchers responsible for enrolling participants were blinded to the allocation sequence, as were those assigning the participants to interventions. This trial followed a triple-blind design, ensuring participants, evaluators, and statisticians remained unaware of the materials being tested. Due to differences in material form and procedural techniques, blinding the operator was not feasible. The two materials were matched for color and exhibited comparable clinical handling, reducing the likelihood of detection by participants. Outcome assessment was conducted by independent evaluators using coded clinical records without any reference to group assignment.

### Clinical procedures

**Fig. 1:**
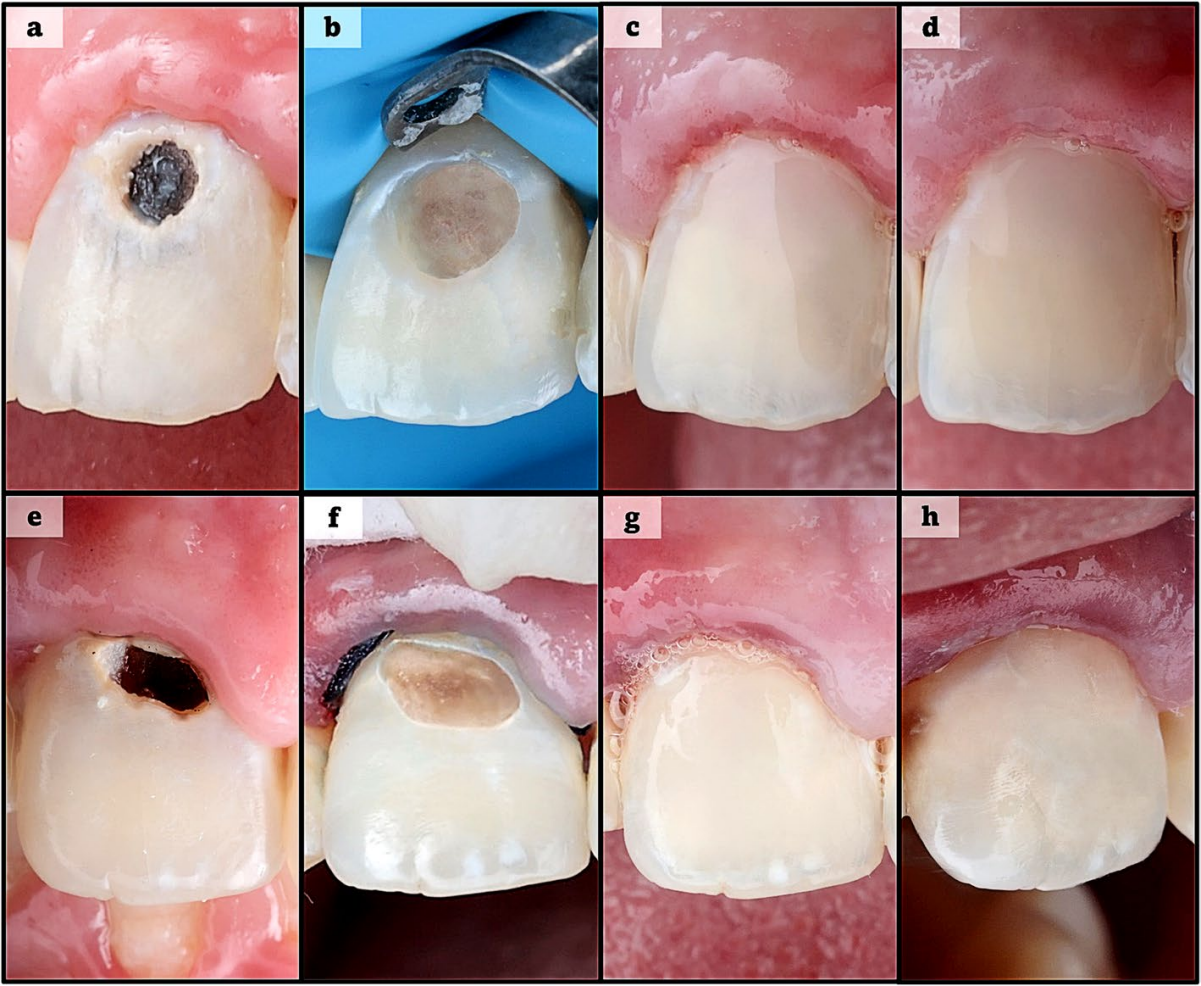
Clinical procedures for each group. Images (**a**–**d**) represent LS-GRC group at preoperative stage, isolation and cavity preparation, restoration, and 12-month follow-up. Images (**e**–**h**) represent RMGI group at corresponding stages. Note surface luster loss in image (**h**), representing RMGI restoration at 12-month follow-up

The materials used are outlined in Table 1 and were placed in accordance with the manufacturers’ instructions. All clinical procedures were performed by a single operator (Fig. 1) with over 10 years of clinical experience in restorative dentistry. Prior to the trial, the operator placed 10 restorations with each material on patients who were not included in the study. These restorations were assessed by two pre-calibrated evaluators, and once each restoration received a score of 1 according to the FDI criteria, the operator was considered calibrated to perform the restorative procedures for the trial.

**Table 1:** Materials’ trade name, classification, composition, manufacturer and lot number

Trade name	Classification	Composition	Manufacturer	Lot number
FineEtch	Etchant	Phosphoric acid (37%)	Spident Co., Incheon, South Korea	FE22133
BeautiBond Xtreme	Universal adhesive	Bisphenol-A-diglycidyl methacrylate (10–20%), Triethyleneglycol dimethacrylate (< 10%), Acid monomer (< 20%), Acetone and water (65–85%), Silane coupling agent (< 5%), Others (< 5%)	Shofu Dental Corporation, Kyoto, Japan	122234
Beautifil II LS	Low-shrinkage giomer resin composite	Aluminofluoro-borosilicate glass (60–70%), Urethane diacrylate (10–20%), Bisphenol A polyethoxy methacrylate (1–5%), Bisphenol-A-diglycidyl methacrylate (1–5%), Triethyleneglycol dimethacrylate (1–5%), Silane coupling agent (1–10%), Silicon Dioxide (1–3%), Aluminum Oxide (1–3%), Reaction initiator, Pigments, Others	Shofu Dental Corporation, Kyoto, Japan	012377
Dentin Conditioner	Mild acidic solution	Polyacrylic acid 10%, Distilled water 90% (by weight)	GC Corporation, Tokyo, Japan	2203041
Fuji II LC capsules	Resin-modified glass ionomer	Distilled water (20%−30%), Polyacrylic acid (20%−30%), 2-hydroxylethyl methacrylate (30%−35%), Urethane dimethacrylate (< 10%), Camphorquinone (< 1%), Fluoroaluminosilicate glass	GC Corporation, Tokyo, Japan	220221B
EQUIA Forte coat	Resin coat	Methyl methacrylate (25–50%), Photoinitiator (1–5%), Synergist (1–5%), Phosphoric acid ester monomer (1–5%), Butylated hydroxytoluene (< 1%)	GC Corporation, Tokyo, Japan	2104221

### Field isolation and cavity preparation

After administering local anesthesia, appropriate isolation techniques were employed according to the restorative material to be applied. For the LS-GRC group, isolation was achieved using rubber dam sheets (Sanctuary Dental Dam, Sanctuary Health Sdn Bhd, Perak, Malaysia), subgingival clamps (KSK Clamps, Dentech Corporation, Tokyo, Japan) and dental floss ligatures. For the RMGI group, moisture control was achieved using cotton rolls, high-volume suction, and retraction cord (Ultrapak E-Knitted Cord, Ultradent Products Inc., Utah, USA).

Cavities were conservatively prepared using pear-shaped, blue-coded diamond burs (Dia-burs, Mani Inc., Tochigi, Japan). Sharp dental excavators (Dentsply, Maillefer, Ballaigues, Switzerland) and carbide round-shaped burs in a low-speed handpiece were used to remove carious dentin. The cavities were refined using yellow-coded, ultrafine diamond burs. For the group receiving LS-GRC, a short incisal bevel was created.

### Restorative procedures

#### Intervention group (LS-GRC)

Selective enamel etching was performed for 15 s (FineEtch 37, Spident Co., Incheon, South Korea), followed by copious rinsing and gentle air drying. A universal adhesive (BeautiBond Xtreme, Shofu Dental Corporation, Kyoto, Japan) was applied, rubbed for 20 s, gently air-thinned for 3 s, followed by a strong air-blow until the surface appeared glossy with no visible movement of the adhesive. The adhesive was photo-polymerized for 10 s using an LED light-curing device (CuringPen, Changzhou Sifary Medical Technology Co., Jiangsu, China). LS-GRC (Beautifil II LS, Shofu Dental Corporation, Kyoto, Japan) was incrementally placed and shaped with titanium-nitride-coated applicators, with each increment not exceeding 2 mm in thickness and photo-polymerized for 20 s, in accordance with the manufacturer’s instructions.

#### Control group (RMGI)

Dentin conditioning was performed for 20 s (Dentin Conditioner, GC Corporation, Tokyo, Japan) followed by copious rinsing and gentle air drying, ensuring a clinically moist surface. The capsule plunger was pressed to activate it, then mixed for 10 s in a high-speed amalgamator (Mix 2000, Carlo De Giorgi, Milano, Italy). The mixture was then incrementally extruded into the prepared cavity and shaped with titanium-nitride-coated applicators, with each increment not exceeding 2 mm in thickness and photo-polymerized for 20 s, in accordance with the manufacturer’s instructions.

### Finishing and polishing

Restoration contouring was performed using yellow-coded diamond burs, followed by finishing and polishing with cups and points (OneGloss, Shofu Dental Corporation, Kyoto, Japan) until an even, glossy surface was achieved. For RMGI, after finishing and polishing, a resin coat (EQUIA Forte Coat, GC Corporation, Tokyo, Japan) was applied and photo-polymerized for 20 s.

### Outcome assessment

The primary outcome was the clinical performance of the restorations, and the secondary outcome was their cost-effectiveness. Restorations were evaluated using the revised FDI criteria [15] by two trained, calibrated and blinded evaluators, each with over 15 years of clinical experience in restorative dentistry. Calibration included reviewing the FDI criteria and a supplementary PowerPoint presentation of clinical photographs illustrating various scoring scenarios. Additional calibration involved reviewing published clinical cases with high-quality images that exemplified the criteria and corresponding scores [15]. Evaluations were conducted at baseline, 6 months and 12 months. In cases of disagreement, a discussion was held to reach consensus. Inter-examiner reliability was assessed using Cohen’s kappa and showed almost perfect agreement (κ = 0.86). The FDI criteria evaluated functional (material fracture and retention, marginal adaptation, form and contour), biological (caries at restoration margins, hard tissue defects, postoperative sensitivity) and esthetic (surface luster and texture, marginal staining, and color match) properties. Restorations were scored as excellent (1), good (2), satisfactory (3), unsatisfactory (4), or poor (5). A score of 1 to 3 indicated an acceptable restoration, a score of 4 indicated an unacceptable restoration but repair was possible, and a score of 5 indicated an unacceptable restoration where repair was not possible or reasonable (Table 2). Cost-effectiveness was assessed by calculating the Incremental Cost-Effectiveness Ratio (ICER) and the Cost per Success Ratio (CPSR). Adverse events, such as pain, sensitivity or restoration failure, were recorded through participant-reported outcomes and clinical examination at each follow-up visit.

**Table 2:** Revised World Dental Federation (FDI) criteria

	**Functional properties (domain F)**		
	**F1: fracture of material and retention**	**F2: marginal adaptation**	**F4: form and contour**
Criteria	*Visual examination and short air drying*	*Visual examination, short air drying, and 250-µm probe*	*Visual examination*
1. Clinically excellent/very good (sufficient)	Restoration is completely present without deficiencies detectable after air drying. No crack, chip- ping/delamination, or material bulk fracture	Ideal marginal adaptation of the restoration at the dental hard tissue after air drying. No marginal gap detectable by gentle probing	Outline, contour, convexity, embrasure, and/or marginal ridges are restored ideally in comparison to the individual, age-related and functional anatomy. No marginal step detectable by gentle probing
2. Clinically good (sufficient)	Restoration is completely present with minor deficiencies detectable after air drying, e.g., insignificant material chipping or one hairline crack	Slight deficiencies of marginal adaptation after air drying. Minor, superficial marginal gap(s) or ditching	Minor deviations in outline, contour, convexity, embrasure, and/or marginal ridges in comparison to the individual, age-related and functional anatomy, AND/OR minor marginal steps, overhangs detectable by gentle probing
3. Clinically satisfactory (sufficient)	Restoration is present with deficiencies detectable without air drying, e.g., hairline cracks or dis- tinct material loss (chipping). Material loss can mainly be corrected by refurbishment if needed	Distinct deficiencies of marginal adaptation without air drying: marginal gap(s) or ditching (width < 250 µm and/or depth < 2 mm)	Outline, contour, convexity, embrasure, and/or marginal ridges are distinctly misshaped but clinically acceptable and/or distinct negative/positive steps, overhangs. Refurbishment (removal of overhangs/steps) to some extent is possible
4. Clinically unsatisfactory (partially insufficient)	Localized but severe deficiencies regarding fracture and retention, e.g., chipping/delamination which cannot be refurbished, bulk fracture, or partially loose/lost restoration. Repair is possible. Lost indirect restoration, which can be recemented/reluted, is considered here	Localized but severe deficiencies of marginal adaptation: width ≥ 250 µm and/or depth ≥ 2 mm marginal gap(s). Partially loose/lost restoration. Repair is possible	Outline, contour, convexity, embrasure, and/or marginal ridges are in parts severely undersized in comparison to the individual, age-related, and functional anatomy AND/OR prominently negative marginal steps. Repair is possible
5. Clinically poor (entirely insufficient)	Generalized severe deficiencies, e.g., extensive delamination, multiple bulk fractures, or (nearly) completely loose/lost restoration. Repair not possible/reasonable	Generalized and severely compromised marginal adaptation: width ≥ 250 µm and/or depth ≥ 2 mm. Complete loose/lost restoration. Repair not possible/reasonable	Outline, contour, convexity, embrasure, and/or marginal ridges are generally and severely under- or oversized in comparison to the individual, age- related, and functional anatomy. Repair not possible/reasonable
	**Biological properties (domain B)**		
	**B1: caries at restoration margin (CAR)**	**B2: dental hard tissue defects at restoration margin**	**B3: postoperative hypersensitivity/pulp status**
Criteria	*Visual examination, short air drying, and 250-µm probe*	*Visual examination*	*Tooth hypersensitivity reported by patient; pulp sensitivity tested with cold stimulus*
1. Clinically excellent/very good (sufficient)	No caries/demineralization at the restoration margin detectable after air drying	Intact dental hard tissue without crack lines and fractures at the restoration margin	No postoperative hypersensitivity or pain on chewing and/or cold/warm food items reported by the patient. Normal (short) reaction to sensitivity test on cold
2. Clinically good (sufficient)	First visible signs of a non-cavitated caries lesion at the restoration margin detectable after air drying	Minor vertical/horizontal hairline crack lines in enamel at the restoration margin	Patient reports minor postoperative hypersensitivity or minor pain on chewing and/or cold/warm food items reported by the patient for a limited period of time (< 1 week). Normal (short) reaction to sensitivity test on cold
3. Clinically satisfactory (sufficient)	Established, non-cavitated caries lesion or microcavity at the restoration margin detectable without air drying	Distinct enamel chipping or enamel fracture at the restoration margin. If necessary, deficiencies can be corrected by refurbishment	Patient reports distinct postoperative hypersensitivity or distinct pain on chewing and/or cold/warm food items reported by the patient for a prolonged period of time (> 1 week). Normal (short) or more intense reaction to sensitivity test on cold
4. Clinically unsatisfactory (partially insufficient)	Localized dentin cavity (width > 250 µm, depth > 2 mm) at the restoration margin. Repair is possible	Severe marginal (enamel) fracture, partially fractured cusp or ridge at the restoration margin. Repair is possible	Patient reports severe/persistent, postoperative hypersensitivity or persistent pain on chewing and/or cold/warm food items reported by the patient for a prolonged period of time (> 1 month) AND/OR intense reaction to sensitivity test on cold
5. Clinically poor (entirely insufficient)	Extensive dentin cavity at the restoration margin. Repair not possible/reasonable	Cusp or tooth fracture, e.g., involving enamel, dentin, and cementum possible with mobile fragments/pain when biting OR cracked tooth syndrome related to restoration. Repair not possible/reasonable	Both symptoms indicate irreversible pulpitis. Endodontic treatment requires access cavity only Irreversible pulpitis, nonvital tooth, pulp necrosis with or without periapical periodontitis after restoration placement. Endodontic treatment requires replacement of the restoration
	**Aesthetic properties (domain A)**		
	**A1: surface luster and surface texture**	**A2: marginal staining**	**A3: color match**
Criteria	*Visual examination and short air drying*	*Visual examination and short air drying*	*Visual examination*
1. Clinically excellent/very good (sufficient)	Surface luster and surface texture comparable to dental hard tissue/adjacent teeth after air drying	No marginal staining detectable after air drying	No deviation in shade, translucency/opacity between restoration, and neighboring dental hard tissue/adjacent teeth
2. Clinically good (sufficient)	Slightly dull surface luster and/or surface texture with minor deviations, e.g., isolated/small marks, pores, and/or voids detectable compared to dental hard tissue/adjacent teeth after air drying	Minor marginal staining detectable after air drying	Minor deviation in shade, translucency/opacity between restoration, and neighboring dental hard tissue/adjacent teeth detectable
3. Clinically satisfactory (sufficient)	Dull surface luster and/or surface texture with distinct deviations, e.g., marks, pores, and/or voids detectable compared to dental hard tissue/adjacent teeth detectable without air drying Refurbishment is possible	Distinct marginal staining detectable without air drying but not displeasing. Refurbishment is possible	Distinct deviation in shade, translucency/opacity between restoration, and neighboring dental hard tissue/adjacent teeth detectable but not displeasing
4. Clinically unsatisfactory (partially insufficient)	Localized, displeasing dull surface luster and/or rough surface texture with substantial deviations/multiple pores/voids detectable compared to dental hard tissue/adjacent teeth which can be repaired	Localized, displeasing deep marginal staining. Marginal staining can be removed/improved by repair	Localized, displeasing deviation in shade, translucency/opacity between restoration, and neighboring dental hard tissue/adjacent teeth which can be improved by repair
5. Clinically poor (entirely insufficient)	Generalized, displeasing dull surface luster and/or rough surface texture with substantial deviations/multiple pores/voids compared to dental hard tissue/adjacent teeth. Repair not possible/reasonable	Generalized, displeasing deep marginal staining. Repair not possible/reasonable	Generalized, displeasing deviation in shade, translucency/opacity between restoration, and neighboring dental hard tissue/adjacent teeth. Repair not possible/reasonable

### Statistical methods

Data were analyzed using MedCalc software, version 22 for Windows (MedCalc Software Ltd., Ostend, Belgium). Categorical data were expressed as frequencies and percentages. Intergroup comparisons were performed using the Chi-Squared test, with statistical significance level set at *P* ≤ 0.05. Intragroup comparisons were performed using the Cochran’s Q test, with statistical significance level set at *P* ≤ 0.016 after Bonferroni correction. Relative risk was calculated to assess clinical significance. Survival rates were analyzed using the Kaplan–Meier method and the Log-rank test. The confidence interval was set at 95% with 80% power, and all tests were two-tailed.

Participants were assigned to groups based on their initial randomization. Missing data were handled through per-protocol analysis, including only those who completed the 12-month follow-up. Participants lost to follow-up were excluded from this analysis.

Incremental Cost-Effectiveness Ratio (ICER) and Cost per Success Ratio (CPSR) were used to compare the cost and clinical outcomes of LS-GRC and RMGI in treating class V cavities. Cost-effectiveness calculations were based on both direct and indirect treatment costs. Direct costs included the material unit price, the armamentarium used, clinical application time, and potential costs related to repair or replacement. Indirect costs considered patient-related factors such as time, transportation, and the number of follow-up visits.

#### Incremental Cost-Effectiveness Ratio (ICER)

The ICER was used to compare the cost-effectiveness of LS-GRC versus RMGI by evaluating the additional cost required for each additional successful outcome achieved with LS-GRC compared to RMGI. The ICER was calculated using the following formula:

$$ICER=\frac{(\text{C}_2-\text{C}_1)}{(\text{E}_2-\text{E}_1)}$$

Where:

- C_2_ and C_1_ are the total costs of LS-GRC and RMGI, respectively,
- E_2_ and E_1_ are the respective effective outcomes, defined as the number of restorations rated as clinically excellent (FDI score = 1) at 12 months.

#### Cost per Success Ratio (CPSR)

The CPSR was calculated to evaluate the cost per clinically successful restoration, determining the cost-effectiveness of each material in achieving successful outcomes, defined as restorations that showed no failure after 12 months. The CPSR was calculated using the following formula:

$$CPSR=\frac{Total\,Cost}{Number\;of\;Successes}$$

Where:

- Total Cost refers to the overall cost per patient for each material,
- Number of Successes refers to the number of restorations rated as clinically excellent (FDI score = 1) at 12 months.

## Results

### Demographic data

**Fig. 2:**
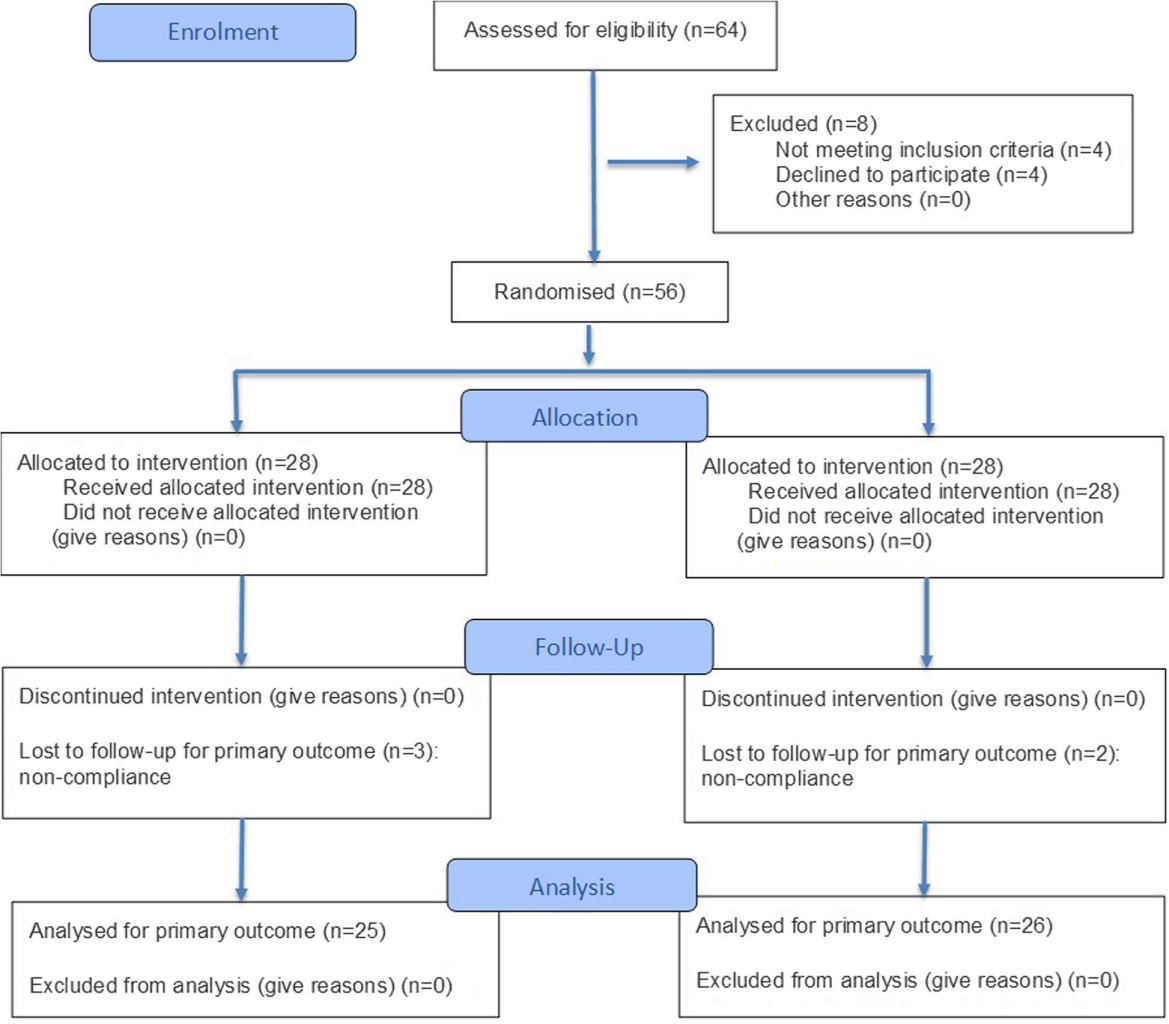
CONSORT 2025 flow diagram

A total of 51 participants completed the 12-month follow-up, resulting in a 91% retention rate (Fig. 2). No statistically significant differences were observed between the two groups regarding age (*P* = 0.560), gender (*P* = 0.7899), or teeth distribution (*P* = 0.8490), as shown in Table 3.

**Table 3:** Participants’ demographic distribution

**Group**	**Age (years)**			**Row total (RT)**
LS-GRC	32.2 ± 7.9			32.8 ± 7.94
RMGI	33.4 ± 8.1			
**Group**	**Gender**			**Row total (RT)**
	**Male**		**Female**	
LS-GRC	13 46.4% RT 52.0% CT		15 53.6% RT 48.4% CT	28 (50%)
RMGI	12 42.9% RT 48.0% CT		16 57.1% RT 51.6% CT	28 (50%)
Column total (CT)	25 (44.6%)		31 (55.4%)	56
*p*-value	*p* = 0.7899			
**Group**	**Teeth**			
	**Maxillary central incisor**	**Maxillary lateral incisor**	**Maxillary** **canine**	**Row total (RT)**
LS-GRC	10 55.6% RT 35.7% CT	9 47.4% RT 32.1% CT	9 47.4% RT 32.1% CT	28 (50%)
RMGI	8 44.4% RT 28.6% CT	10 52.6% RT 35.7% CT	10 52.6% RT 35.7% CT	28 (50%)
Column total (CT)	18 (32.1%)	19 (33.9%)	19 (33.9%)	56
*p*-value	*p* = 0.8490			

### Clinical performance

After 12 months, intergroup comparisons revealed no significant differences for most outcomes (*P* > 0.05), except for surface luster, where LS-GRC showed superior results (*P* < 0.05). Intragroup comparisons revealed no significant changes for LS-GRC (*P* < 0.016), while significant changes were observed for RMGI (*P* < 0.016) in surface luster (Table 4).

**Table 4:** Frequency and percentage of FDI scores comparing groups across parameters and follow-up periods

Outcome	Follow-up	LS-GRC						RMGI						p-value
		**N**	**Success**			**Failure**		**N**	**Success**			**Failure**		
			**1**	**2**	**3**	**4**	**5**		**1**	**2**	**3**	**4**	**5**	
Fracture of material and retention	Baseline	28	28(100%)	0(0%)	0(0%)	0(0%)	0(0%)	28	28(100%)	0(0%)	0(0%)	0(0%)	0(0%)	*p* = 1.0000
	6 months	26	26(100%)	0(0%)	0(0%)	0(0%)	0(0%)	28	28(100%)	0(0%)	0(0%)	0(0%)	0(0%)	*p* = 0.7855
	12 months	25	25(100%)	0(0%)	0(0%)	0(0%)	0(0%)	26	26(100%)	0(0%)	0(0%)	0(0%)	0(0%)	*p* = 0.8886
	*p*-value		*p* = 0.097						*p* = 0.135					
Marginal adaptation	Baseline	28	28(100%)	0(0%)	0(0%)	0(0%)	0(0%)	28	28(100%)	0(0%)	0(0%)	0(0%)	0(0%)	*p* = 1.0000
	6 months	26	26(100%)	0(0%)	0(0%)	0(0%)	0(0%)	28	28(100%)	0(0%)	0(0%)	0(0%)	0(0%)	*p* = 0.7855
	12 months	25	25(100%)	0(0%)	0(0%)	0(0%)	0(0%)	26	26(100%)	0(0%)	0(0%)	0(0%)	0(0%)	*p* = 0.8886
	*p*-value		*p* = 0.097						*p* = 0.135					
Form and contour	Baseline	28	28(100%)	0(0%)	0(0%)	0(0%)	0(0%)	28	28(100%)	0(0%)	0(0%)	0(0%)	0(0%)	*p* = 1.0000
	6 months	26	26(100%)	0(0%)	0(0%)	0(0%)	0(0%)	28	28(100%)	0(0%)	0(0%)	0(0%)	0(0%)	*p* = 0.7855
	12 months	25	25(100%)	0(0%)	0(0%)	0(0%)	0(0%)	26	26(100%)	0(0%)	0(0%)	0(0%)	0(0%)	*p* = 0.8886
	*p*-value		*p* = 0.097						*p* = 0.135					
Caries at restoration margin	Baseline	28	28(100%)	0(0%)	0(0%)	0(0%)	0(0%)	28	28(100%)	0(0%)	0(0%)	0(0%)	0(0%)	*p* = 1.0000
	6 months	26	26(100%)	0(0%)	0(0%)	0(0%)	0(0%)	28	28(100%)	0(0%)	0(0%)	0(0%)	0(0%)	*p* = 0.7855
	12 months	25	25(100%)	0(0%)	0(0%)	0(0%)	0(0%)	26	26(100%)	0(0%)	0(0%)	0(0%)	0(0%)	*p* = 0.8886
	*p*-value		*p* = 0.097						*p* = 0.135					
Dental hard tissue defects at restoration margin	Baseline	28	28(100%)	0(0%)	0(0%)	0(0%)	0(0%)	28	28(100%)	0(0%)	0(0%)	0(0%)	0(0%)	*p* = 1.0000
	6 months	26	26(100%)	0(0%)	0(0%)	0(0%)	0(0%)	28	28(100%)	0(0%)	0(0%)	0(0%)	0(0%)	*p* = 0.7855
	12 months	25	25(100%)	0(0%)	0(0%)	0(0%)	0(0%)	26	25(96.2%)	1(3.8%)	0(0%)	0(0%)	0(0%)	*p* = 0.3268
	*p*-value		*p* = 0.097						*p* = 0.050					
Postoperative hypersensitivity	Baseline	28	28(100%)	0(0%)	0(0%)	0(0%)	0(0%)	28	28(100%)	0(0%)	0(0%)	0(0%)	0(0%)	*p* = 1.0000
	6 months	26	26(100%)	0(0%)	0(0%)	0(0%)	0(0%)	28	28(100%)	0(0%)	0(0%)	0(0%)	0(0%)	*p* = 0.7855
	12 months	25	25(100%)	0(0%)	0(0%)	0(0%)	0(0%)	26	26(100%)	0(0%)	0(0%)	0(0%)	0(0%)	*p* = 0.8886
	*p*-value		*p* = 0.097						*p* = 0.135					
Surface luster and texture	Baseline	28	28(100%)	0(0%)	0(0%)	0(0%)	0(0%)	28	28(100%)	0(0%)	0(0%)	0(0%)	0(0%)	*p* = 1.0000
	6 months	26	26(100%)	0(0%)	0(0%)	0(0%)	0(0%)	28	28(100%)	0(0%)	0(0%)	0(0%)	0(0%)	*p* = 0.7855
	12 months	25	25(100%)	0(0%)	0(0%)	0(0%)	0(0%)	26	14(53.8%)	12(46.2%)	0(0%)	0(0%)	0(0%)	*p* = 0.0001*
	*p*-value		*p* = 0.097						*p* < 0.001*					
Marginal staining	Baseline	28	28(100%)	0(0%)	0(0%)	0(0%)	0(0%)	28	28(100%)	0(0%)	0(0%)	0(0%)	0(0%)	*p* = 1.0000
	6 months	26	26(100%)	0(0%)	0(0%)	0(0%)	0(0%)	28	28(100%)	0(0%)	0(0%)	0(0%)	0(0%)	*p* = 0.7855
	12 months	25	23(92%)	2(8%)	0(0%)	0(0%)	0(0%)	26	26(100%)	0(0%)	0(0%)	0(0%)	0(0%)	*p* = 0.1452
	*p*-value		*p* = 0.022						*p* = 0.135					
Color match	Baseline	28	28(100%)	0(0%)	0(0%)	0(0%)	0(0%)	28	28(100%)	0(0%)	0(0%)	0(0%)	0(0%)	*P* = 1.0000
	6 months	26	26(100%)	0(0%)	0(0%)	0(0%)	0(0%)	28	28(100%)	0(0%)	0(0%)	0(0%)	0(0%)	*P* = 0.7855
	12 months	25	25(100%)	0(0%)	0(0%)	0(0%)	0(0%)	26	26(100%)	0(0%)	0(0%)	0(0%)	0(0%)	*P* = 0.8886
	*P* value		*P* = 0.097						*P* = 0.135					

^*^Significant; Chi-Squared test for intergroup comparisons (*p* ≤ 0.05); Cochran’s Q test for intragroup comparisons (*p* ≤ 0.016, Bonferroni-adjusted)

In the survival analysis, no restorations failed (received a score of 4 or 5 according to the FDI criteria, indicating an unacceptable restoration requiring replacement) in either group after 12 months. Two restorations in the LS-GRC group received a score of 2 (clinically good) due to marginal staining, while twelve restorations in the RMGI group scored 2 for surface luster and texture. Accordingly, LS-GRC demonstrated 92% clinically excellent restorations, whereas RMGI exhibited 53.8% clinically excellent restorations. However, all restorations were considered clinically successful. The proportion of restorations rated as clinically good (score 2) was 83% lower for LS-GRC compared to RMGI after 12 months (95% CI: 0.04306 to 0.6978, *P* = 0.0136). Kaplan–Meier survival curves and Log-rank test revealed a statistically significant difference between the two groups (*P* = 0.002225).

### Cost-effectiveness

The ICER analysis showed that the cost for each additional 1% improvement in clinical outcomes with LS-GRC was approximately 1.6 times higher than that of RMGI. The CPSR value for LS-GRC was 4.6% lower than that of RMGI, suggesting that while LS-GRC incurs a higher upfront cost, it may offer better value over time. In other words, despite the higher initial cost, LS-GRC demonstrates comparable clinical performance to RMGI, with significantly improved esthetic surface quality and favorable cost-effectiveness over the 12-month period.

## Discussion

Restoring cervical carious lesions presents a complex challenge in adhesive dentistry, primarily due to the absence of enamel margins, the high C-factor, and cyclic flexural stresses [16, 17]. These factors often compromise the marginal seal and lead to clinical issues, such as discoloration, microleakage, secondary caries, and postoperative sensitivity [18, 19]. Giomers, glass ionomers, and resin composites are commonly used in class V cavities for their beneficial properties [20]. Although clinical evaluations of S-PRG restorative materials remain limited, in vitro studies have shown promising results [21]. This trial is the first to compare the clinical performance and cost-effectiveness of LS-GRC and RMGI in treating cervical carious lesions.

Rubber dam isolation was used only in the LS-GRC group due to the material’s handling characteristics and moisture sensitivity. The RMGI group was treated under cotton roll isolation, in line with manufacturer recommendations, which discourage dentin desiccation. Water is essential for initiating the acid–base reaction critical to the setting and adhesion of glass ionomers. Adhering to manufacturer guidelines reflects real-world clinical practice and enhances the accuracy of cost-effectiveness analysis, as isolation methods impact both procedural time and armamentarium costs. While saliva contamination has been shown to impair the dentin bond strength of adhesives, RMGI bonding remains unaffected [22]. Additionally, GI-based materials are prone to crack formation and dissolution when placed in overly dry environments, potentially compromising their longevity [22, 23]. Clinical studies support that cotton roll isolation provides adequate moisture control for RMGIs while reducing treatment time, contributing to cost-effectiveness [22, 24]. These handling differences highlight the importance of tailoring isolation to the specific requirements of each material in order to optimize both clinical and economic outcomes.

Similarly, cavity preparation was customized based on the adhesive requirements of each material. Enamel beveling was performed only in the LS-GRC group to enhance micromechanical retention by removing aprismatic enamel, increasing bonding surface area, and improving acid etching and surface wetting—factors that reduce polymerization shrinkage stress and microleakage [25, 26]. Beveling improves enamel reactivity by exposing enamel prisms, thereby enhancing bonding performance [27]. Beveling also enhances esthetics by blending the restoration with the surrounding tooth structure, improving color integration and minimizing marginal visibility [25, 26]. These benefits are particularly relevant to resin composites, and many studies have routinely incorporated beveling into their clinical protocols for resin composite restorations [24–26, 28–33]. In contrast, beveling was omitted in the RMGI group consistent with common clinical guidelines, as RMGIs rely more on chemical adhesion than micromechanical interlocking [34, 35]. Tailoring preparation protocols to material properties supports clinical effectiveness and real-world restorative applicability.

Fracture and retention results in the LS-GRC group align with previous research [36–38] demonstrating acceptable functional performance. LS-GRC’s formulation minimizes polymerization shrinkage and associated stress through its proprietary monomer and high-density pre-polymerized fillers [2]. Additionally, the presence of carboxylic and phosphonic acid monomers in the adhesive enables strong chemical bonding with the tooth structure, improving resistance to water degradation and promoting long-term stability of the adhesive interface [5]. These features are likely to contribute to the positive outcomes observed in retention and fracture resistance within the intervention group. In the control group, retention of RMGI is credited to its two-fold adhesion; chemical bonding to the tooth via ionic interactions, and micromechanical bonding through a resin-dentin hybrid layer [39]. Furthermore, the low elastic modulus of RMGI allows it to flex under occlusal load, reducing stress and risk of debonding [40]. Our findings are consistent with several studies [10, 34, 35, 41] that reported no restoration losses due to the durable adhesion of RMGI.

Concerning marginal adaptation and staining, the findings in LS-GRC group can be attributed to the material’s low tendency for volumetric shrinkage and the associated stress. This is due to the unique Steric Repulsion Structured (SRS) molecule, which reduces shrinkage through molecular steric repulsion, contributing to a strong and stable restoration microstructure [2, 36]. These results are consistent with previous studies [36–38], which reported clinically successful LS-GRC restorations with acceptable marginal integrity over follow-up periods of up to three years. However, an invitro study [42] reported compromised marginal integrity in giomer resin composite compared to resin-modified glass ionomer in class V cavities. The authors attributed this to the absence of enamel pre-etching prior to the application of a self-etch adhesive, which may have been insufficient for effective enamel bonding. In contrast, the present study employed selective enamel etching, potentially enhancing adhesion. Furthermore, differences in study type and materials may also explain the discrepancy in findings. While in vitro studies provide valuable preliminary insights, they may not fully replicate the clinical performance of restorative materials under intraoral conditions. Additionally, the previous study used a conventional giomer resin composite, whereas the current study utilized a low-shrinkage variant, which may offer better marginal adaptation. Research suggests that improved marginal outcomes may be achieved with low-shrinkage and injectable giomers, particularly in high C-factor cavities [4]. This is further supported by a study [34] demonstrating superior marginal adaptation with injectable giomer resin composites, highlighting the benefits of newer injectable and low-shrinkage formulations in minimizing marginal discrepancies.

RMGI has been reported to exhibit a lower tendency for microleakage, contributing to superior marginal integrity due to its chemical adhesion and flowable nature. This is further enhanced by dentin pre-treatment with a conditioner, which promotes ion diffusion into the partially demineralized substrate and improves adhesion through the acid-resistant dissolution of the tooth-GIC interaction phase [19, 43, 44]. Our findings agree with several studies [34, 35, 41] that reported favorable marginal integrity of RMGI restorations. However, other studies [10, 14] observed marginal discoloration, and increased Bravo scores after 36 months, likely attributable to the extended follow-up period.

Regarding surface luster and texture, a statistically significant difference was observed between groups, with RMGI showing a noticeable increase in score (2) after 12 months. Surface quality is critical to clinical success, as a smooth surface inhibits plaque accumulation and discoloration, thereby enhancing the clinical performance of cervical restorations [17]. These surface characteristics are influenced by factors such as filler size, wear resistance, and the material’s ability to maintain its surface finish and polish. Larger particle sizes likely contribute to increased surface roughness, which may explain why LS-GRC, with a mean particle size of 0.4µm [45], exhibited a smoother surface compared to RMGI, which has a mean particle size of 25 µm [46]. Additionally, the rougher surface of RMGI may be attributed to its heterogeneous structure, where larger filler particles are embedded in a soft matrix. This can lead to filler particle dislodgement due to the weak cohesion between the polyalkenoate and poly-HEMA matrices and the glass particles. A previous study [47] illustrated the loss of surface luster in RMGI compared to the maintained luster in LS-GRC, is likely due to the higher water sorption tendency of RMGI, which leads to surface deterioration. Our results are consistent with studies [10, 35, 41] that reported increased roughness and loss of luster in RMGI, attributed to its lower abrasion resistance. Other studies [36, 37] confirmed high surface luster achieved and maintained in LS-GRC restorations, owing to their high wear resistance.

Regarding caries at the restoration margins, both materials exhibit bioactivity, contributing to secondary caries prevention [7, 43]. Both giomers and RMGIs possess the unique ability to not only release but also recharge fluoride, thereby providing continual protection to the tooth by decreasing the acid production of cariogenic bacteria, exerting an anti-plaque effect, and reducing tooth mineral solubility. Giomers, through proprietary S-PRG technology, actively release six beneficial ions that inhibit bacterial adhesion, stabilize oral pH, reduce the risk of secondary caries, and promote remineralization [21]. Our findings align with previous clinical studies that concluded no recurrent caries around giomer restorations [4, 36–38]. Similarly, our results are consistent with several other studies [10, 34, 35] that observed no secondary caries surrounding RMGI restorations.

Regarding postoperative sensitivity, the favorable results observed with LS-GRC can be attributed to the use of a universal adhesive in self-etch mode combined with selective enamel etching. This technique avoids acid etching of dentin, thereby reducing the risk of hypersensitivity [48]. Our results are in accordance with previous clinical studies [4, 36–38], where giomer restorations showed no signs of sensitivity. Similarly, RMGI did not exhibit any postoperative sensitivity, likely due to the polyacrylic acid conditioner used, which acts as a surface-modifying agent. This conditioner forms a shallow, micro-porous hybrid layer without unplugging the dentinal tubules, similar to the self-etching protocol [14]. Our findings agreed with studies [10, 34, 35] where no signs of sensitivity or discomfort were observed with RMGI restorations.

Since no previous research has assessed the cost-effectiveness of LS-GRC compared to RMGI, this study provides valuable insights. The cost-effectiveness analysis revealed that LS-GRC has a higher initial cost than RMGI. However, the long-term deterioration of RMGI may require refurbishment or even repair, leading to additional costs. As mentioned earlier, surface quality plays a crucial role, particularly in anterior esthetic restorations. Therefore, despite the higher initial cost of LS-GRC, it may offer long-term durability in restorations.

As far as we know, this clinical trial is the first to assess the performance and cost-effectiveness of the novel LS-GRC in comparison to RMGI for treating cervical carious lesions over 12 months. However, certain limitations exist, including a relatively small sample size and a 12-month follow-up, which may not adequately represent longevity. Larger-scale clinical studies with extended follow-up are necessary to confirm these results and evaluate long-term outcomes. Additionally, further research comparing LS-GRC with other restorative materials in varied clinical settings is recommended.

## Conclusions

Within the limitations of this study, LS-GRC and RMGI exhibited comparable performance and were deemed clinically acceptable in restoring cervical carious cavities after 12 months. Although LS-GRC has a higher initial cost, it offers comparable clinical outcomes to RMGI, with significantly improved esthetic surface quality and favorable cost-effectiveness over the 12-month period. LS-GRC is a promising restorative material that may help achieve optimal cervical restorations with improved clinical success, durability, and sustained cost-effectiveness.

## Data Availability

No datasets were generated or analysed during the current study.
